# Pilot Study on the Utility and Feasibility of a House-Call Checkup of the Medicine Cabinet

**DOI:** 10.3390/pharmacy6030074

**Published:** 2018-07-24

**Authors:** Lore Janssen, Luc Pieters, Hans De Loof

**Affiliations:** 1 Natural Products and Food Research and Analysis (NatuRA), Department of Pharmaceutical Sciences, University of Antwerp, B-2610 Antwerp, Belgium; lore.janssen@student.uantwerpen.be (L.J.); Luc.Pieters@uantwerpen.be (L.P.); 2 Laboratory of Physiopharmacology, University of Antwerp, Universiteitsplein 1, B-2610 Antwerp, Belgium

**Keywords:** medicine cabinet, house-call, refrigeration, medication safety, medication literacy, expiration date

## Abstract

The storage at home of medicines is a poorly researched topic, but it can be a major source of medication errors and other unsafe practices. In this pilot-study, we wanted to get an idea of the scope of the problem and research the feasibility and acceptability of a home-based intervention by a pharmacist. In a convenience sample of 48 households, we encountered numerous problems in a sizable percentage of households. Medicines were frequently not stored out of reach of children, usage instructions and indications were unknown, organization was absent, and there were a plethora of expired medicines present. Refrigeration was less of a problem. Acceptability and perception of utility of the intervention were generally very high. We developed a protocol-based intervention to be used in future research to increase the safe use of medicines at home.

## 1. Introduction

It is safe to assume that there are problems with the storage of medications at home and there is little doubt that a large proportion of stored unused medicines are beyond the expiration date. There are also questions about correct atmospheric conditions, repackaging, labeling and organization of the medicine cabinet. It is clear from the study of takeback events or waste-disposal statistics and other studies that large quantities of medicines are stored at home that can be a source of unsafe medication use [1,2,3,4,5,6,7]. These studies reported the prominent presence of analgesics including NSAIDs [2,4] and opioids [2,7], but “leftover” antibiotics were also ubiquitous [3]. Evidently, many medicines present were expired [2,4]. We initiated a pilot study to further delineate the nature and the extent of the problem and to study the utility and acceptability of a house-call by a pharmacist. We focused on some simple organizational aspects of the storage at home: place, temperature, out of reach of children, orderliness, packaging, and leaflets. We hypothesize that, if acceptable, this intervention could reduce the public health threat caused by unsafe medicine use.

## 2. Materials and Methods

A convenience sample of 48 households was selected for our study. People were contacted in the pharmacy, through social media or were referred to us by people who had participated in the trial. House calls were conducted in March and April of 2017 by L.J., a last year pharmacy-student.

An initial checklist/questionnaire was used involving location, ordering, children’s access possibility, expiration dates, original packaging, knowledge of the kind of product/indication, and temperature/refrigeration necessity. A bathroom, being too humid, was not considered an appropriate place. A dry place without direct sunlight and within correct temperature range was deemed appropriate. For people on chronic medication, the presence of a medication scheme/plan was also looked for, and if this scheme was comprehensible to them. 

At the end of the visit, three documents were handed out. A brief reminder of the aim of the study, together with contact information of the pharmacist in case there were additional questions, a leaflet about sorting/disposal of medication beyond its expiration date, and a general leaflet about medication storage at home.

## 3. Results

A wide variety of households was sampled: In 12 the maximum age of the head of household was 35 years; in 24 families this was between 35 and 65 years, and in 12 households people above 65 years were present. Of the 28 households with children, 10 families had children younger than 6 years. 

An overview of the results is given in Table 1. 

In most cases (92%) medicines were stored separately. The storage location was badly chosen in a substantial number of households: In 38% it was not out of reach of children. Even in households with small children, this figure reached 20%. In contrast, the medicine cabinet of elderly people was structured in a more orderly fashion (82%) compared to those households with small children (50%).

Refrigeration was less of a problem; the vast majority of those medicines were stored correctly (90%), but in a substantial number of households (23%), medicines that do not need to be stored there were still found in the refrigerator. 

**Figure 1:**
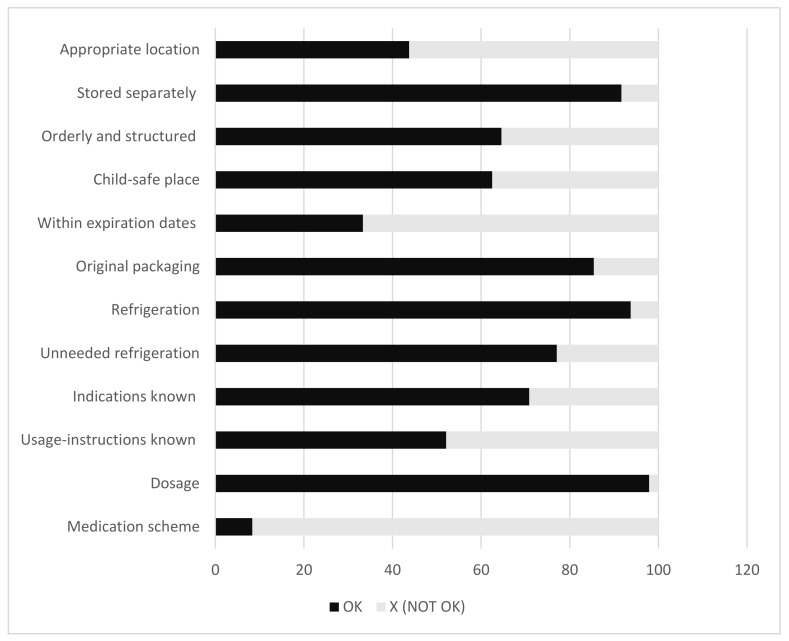
Characteristics (in percentages) of the household medicine cabinets.

In 15% of medicine cabinets, medicines were not stored in their original container and, not unexpectedly, in two-thirds of households across our subgroups, expired medicines were present. A graphical representation of the results is shown in Figure 1.

Slightly more than half of the medicines had extra labeling, but in about 30%, the indication was unknown, and in nearly half of the cases, exact usage instructions were forgotten although dosage recall was good. A medication scheme was only present in a small number of households.

## 4. Discussion and Conclusions

The data from this initial pilot study confirm the hypothesis that a lot of improvement is possible in how medications are stored at home. Child safety is certainly inadequate; taking into account that the majority of accidents leading to emergency department visits originate from unsupervised ingestions by children accessing medications on their own [8], big security gains are certainly possible. This also holds true for households where children are present only occasionally (e.g., grandparents). 

The other major safety problem is the pervasive lack of knowledge about the medicines themselves as indications have been forgotten, and the utilization instructions are no longer known. Although this study is too small to accurately quantify this lack of knowledge, it is nevertheless safe to conclude this illiteracy is hazardous. It is, therefore, not unexpected that there is a lack of structure as people have a hard time classifying the medicines. This is in accordance with other research in which we have shown that in the general population, knowledge about the names of even basic OTC pain medication is often problematic [9].

The prevalence of medicines beyond their expiration date was, as expected, very high. This is indicative of the lack of periodic review of the content and ‘spring cleaning’ as advocated by a number of organizations [10,11]. It is this latter aspect, the lack of organization, that is much more worrisome than the toxicological risks induced by the aging process of the medicines [12]. 

Because of these large deficits in basic knowledge and organization of the medicines involved, the proposed checklist includes more questions about medicines-use than originally planned (see last section of the Appendix A). It can be safely assumed that this service can be the preamble of a full mediation review: Determining for whom a full medication review is indeed warranted and introducing this kind of service to a target population not yet accustomed to this service or its benefits [13]. 

Although the satisfaction of the people whose medicine cabinet received a checkup was not formally queried, most people clearly expressed their gratitude for the service delivered. A substantial number of subjects in the study forwarded addresses to the researcher because they thought those people might also benefit. It should be mentioned that this was a free service and future research should quantify in more detail the time involved to estimate the cost of this kind of intervention. Another limitation of this study is the semi-rural setting in which it was conducted, a place where nearly everybody knows the local pharmacist-mentor personally. In the anonymous setting of a big city, people might be less willing to let a stranger enter their home to go through their medicine cabinet. This obviously limits the generalizability of this intervention, and a formal acceptability assessment may be needed in other settings. However, we do not anticipate major differences in the organizational aspect of the medicine cabinet of people living in an urbanized area. 

Further research into this type of service seems warranted and should provide insights into its effectiveness in reducing unsafe medication use, intoxications, and waste. Our hypothesis can thus be reformulated: Can implementing this service have an impact on public health? We can also safely conclude that the topic of how medicines are stored should receive much more attention when dispensing medicines in the pharmacy, and campaigns to increase awareness of this topic are warranted.

## Figures and Tables

**Table 1:** Overview of the problems encountered in the medicine cabinets.

Sample			Location				Storage				Use			
House-Call Number	Age ^†^	Children	Appropriate Location	Stored Separately	Orderly and Structured ^∆^	Child-Safe Place	Within Expiration Dates	Original Packaging	Refrigeration	Unneeded Refrigeration	Indications Known ^§^	Usage-Instructions Known ^§^	Dosage Known	Medication Scheme
1	3	C	X	OK	OK	OK	X	OK	OK	OK	OK	OK+	OK	X
2	3	C	X	X	OK	OK	X	OK	OK	OK	OK+	OK+	OK	X
3	2	B	OK	OK	X	X	X	OK	OK	OK	X	OK+	OK	X
4	2	B	X	OK	OK	OK	X	OK	OK	X	OK+	X	OK	X
5	2	B	OK	OK	X	OK	X	OK	OK	X	X	OK+	OK	X
6	3	C	X	OK	X	OK	X	OK	OK	OK	X	X	OK	X
7	2	B	OK	OK	OK−	X	X	OK	OK	OK	OK+	X	OK	X
8	3	C	OK	OK	OK−	X	X	X	X	OK	OK	OK	OK	X
9	2	C	X	X	OK−	OK	OK	OK	OK	X	OK	OK+	OK	X
10	3	C	X	OK	OK−	X	OK	OK	OK	OK	OK+	X	OK	X
11	3	C	X	OK	X	X	X	OK	OK	OK	X	X	OK	X
12	1	C	OK	OK	X	OK	OK	X	OK	OK	OK	OK+	OK	X
13	1	A	OK	OK	OK	OK	X	OK	OK	OK	OK	OK+	OK	X
14	1	A	OK	OK	OK−	X	OK	X	OK	OK	OK	OK+	OK	X
15	2	C	OK	OK	OK−	OK	OK	OK	OK	OK	OK	OK+	OK	X
16	2	A	X	X	X	OK	X	OK	OK	OK	X	X	OK	X
17	1	A	OK	OK	X	OK	X	OK	X	OK	OK	X	OK	X
18	2	B	OK	OK	OK	X	OK	OK	OK	X	OK	OK	OK	OK
19	1	A	OK	OK	OK	OK	X	OK	OK	OK	OK	OK+	OK	X
20	2	B	OK	OK	X	OK	X	OK	OK	OK	X	X	OK	X
21	1	C	X	OK	X	OK	X	OK	OK	OK	X	X	OK	X
22	2	B	X	OK	OK	X	X	OK	OK	OK	X	X	OK	X
23	3	C	X	OK	OK	X	X	OK	OK	OK	X	X	OK	OK
24	1	A	X	OK	OK	X	X	OK	OK	X	OK	OK	OK	X
25	1	B	OK	OK	X	OK	OK	X	OK	OK	X	X	OK	X
26	2	C	X	OK	X	X	X	OK	OK	OK	OK+	X	OK	X
27	2	C	X	OK	OK−	X	OK	OK	OK	OK	X	X	X	X
28	3	C	X	OK	OK	OK	OK	OK	OK	OK	X	OK+	OK	X
29	2	B	X	OK	X	OK	X	X	OK	OK	X	X	OK	X
30	3	C	OK	OK	OK	X	X	X	OK	X	OK	OK	OK	OK
31	3	C	X	OK	OK−	OK	X	OK	OK	OK	OK+	X	OK	X
32	2	B	OK	OK	X	OK	X	OK	OK	X	OK+	X	OK	X
33	2	B	X	OK	OK	X	OK	OK	OK	X	OK	OK+	OK	X
34	2	B	X	OK	X	X	OK	OK	OK	OK	OK	OK+	OK	X
35	2	B	OK	OK	OK	OK	OK	OK	OK	OK	OK	X	OK	X
36	3	C	OK	OK	OK−	X	OK	OK	OK	OK	OK+	OK+	OK	OK
37	2	C	X	OK	X	OK	X	OK	OK	X	OK+	X	OK	X
38	1	A	OK	OK	OK	OK	X	OK	OK	OK	OK	OK	OK	X
39	2	B	X	OK	OK−	OK	X	OK	OK	OK	OK+	OK	OK	X
40	1	C	X	OK	X	X	X	OK	OK	OK	OK+	X	OK	X
41	2	A	OK	OK	OK	OK	X	OK	OK	OK	OK	OK	OK	X
42	3	C	X	OK	OK−	OK	X	OK	OK	X	OK	X	OK	X
43	2	B	X	OK	OK	OK	OK	OK	OK	OK	OK	OK	OK	X
44	2	B	X	OK	OK	X	X	OK	OK	OK	OK	OK	OK	X
45	2	B	X	OK	OK	OK	OK	OK	OK	OK	OK	OK+	OK	X
46	1	A	OK	OK	X	OK	OK	OK	OK	OK	OK+	X	OK	X
47	1	A	X	X	OK−	OK	X	OK	X	X	X	X	OK	X
48	2	B	OK	OK	OK−	OK	X	X	OK	OK	OK	OK	OK	X

^†^ age (years) 1: <35, 2: 35–65, 3: >65; children age (years) A: ≤6, B: >6, C: none; ^∆^ OK− ordering was underway; ^§^ OK+ written note on box.

## Appendix A

Checklist

**Question/Statement**
**Location**
Where are medicines stored at home?
What kind of storage is used?
Are medicines stored in an orderly/structured way?
Is the medication kept out of the reach of children?
**Refrigeration**
Are medicines needing refrigeration present?
Are medicines that do not need refrigeration present in the refrigerator?
**Medication**
Are expired medicines present?
Are the products stored in their original packaging?
Are the indications of the medicines known?
Are you familiar about how the medication is used?
**Medication scheme/plan**
Are the patients aware what medicine should be taken at what moment?
Is a Medication scheme/plan present?
